# Predicting the strengths of date fiber reinforced concrete subjected to elevated temperature using artificial neural network, and Weibull distribution

**DOI:** 10.1038/s41598-023-45462-z

**Published:** 2023-10-30

**Authors:** Musa Adamu, Khalil Ur Rehman, Yasser E. Ibrahim, Wasfi Shatanawi

**Affiliations:** 1 Engineering Management Department, College of Engineering, Prince Sultan University, 11586 Riyadh, Saudi Arabia; 2 Department of Mathematics and Sciences, College of Humanities and Sciences, Prince Sultan University, 11586 Riyadh, Saudi Arabia; 3 Department of Mathematics, Air University, PAF Complex E-9, Islamabad, 44000 Pakistan; 4 Department of Medical Research, China Medical University Hospital, China Medical University, Taichung, 40402 Taiwan; 5 Department of Mathematics, Faculty of Science, The Hashemite University, P.O. Box 330127, Zarqa, 13133 Jordan

**Keywords:** Civil engineering, Composites

## Abstract

Date palm fiber (DPF) is normally used as fiber material in concrete. Though its addition to concrete leads to decline in durability and mechanical strengths performance. Additionally, due to its high ligno-cellulose content and organic nature, when used in concrete for high temperature application, the DPF can easily degrade causing reduction in strength and increase in weight loss. To reduce these effects, the DPF is treated using alkaline solutions. Furthermore, pozzolanic materials are normally added to the DPF composites to reduce the effects of the ligno-cellulose content. Therefore, in this study silica fume was used as supplementary cementitious material in DPF reinforced concrete (DPFRC) to reduce the negative effects of elevated temperature. Hence this study aimed at predicting the residual strengths of DPFRC enhanced/improved with silica fume subjected to elevated temperature using different models such as artificial neural network (ANN), multi-variable regression analysis (MRA) and Weibull distribution. The DPFRC is produced by adding DPF in proportions of 0%, 1%, 2% and 3% by mass. Silica fume was used as partial substitute to cement in dosages of 0%, 5%, 10% and 15% by volume. The DPFRC was then subjected to elevated temperatures between 200 and 800 °C. The weight loss, residual compressive strength and relative strengths were measured. The residual compressive strength and relative strength of the DPFRC declined with addition of DPF at any temperature. Silica fume enhanced the residual and relative strengths of the DPFRC when heated to a temperature up to 400 °C. To forecast residual compressive strength (RCS) and relative strength (RS), we provide two distinct ANN models. The first layer's inputs include DPF (%), silica fume (%), temperature (°C), and weight loss (%). The hidden layer is thought to have ten neurons. M-I is the scenario in which we use RCS as an output, whereas M-II is the scenario in which we use RS as an output. The ANN models were trained using the Levenberg–Marquardt backpropagation algorithm (LMBA). Both neural networking models exhibit a significant correlation between the predicted and actual values, as seen by their respective R = 0.99462 and R = 0.98917. The constructed neural models M-I and M-II are highly accurate at predicting RCS and RS values. MRA and Weibull distribution were used for prediction of the strengths of the DPFRC under high temperature. The developed MRA was found to have a good prediction accuracy. The residual compressive strength and relative strength followed the two-parameter Weibull distribution.

## Introduction

Concrete can be used in structures that are exposure to both normal and sever conditions. For normal conditions, there is not much challenge in using the concrete. However, for exposure conditions such as acidic and salt environment, elevated temperature, oil and gas environment, there is need for careful selection of the constituent materials for producing the concrete in such a way that its durability performance is not significantly affected. Even with these, concrete has more advantages over other building materials when used in areas of high exposure conditions. For example, when steel is used in areas of alkaline or chlorine environment, there is high risk of corrosion compared to when reinforced concrete is used. Similarly, in high temperature exposure, plastics, steel and timber tends to loss their structural integrity faster than concrete^1,2^. Even with concrete, long-time exposure to high temperature causes significant loss in structural integrity and strength. Furthermore, spalling in the concrete structure due occur due to induced chemical reactions and physical changes in the concrete’s microstructure when exposed to high temperature for a long period.. When concrete spall, this will significantly affect the ultimate limit and serviceability limit states of the structure and results to failure of the structure. This will have a significant effect on the durability and design life of the structure^3,4^. Therefore, it is necessary to comprehend the relationship between high temperature exposure and the properties of concrete such as durability, mechanical strengths, toughness, and brittleness. This will guide in detecting and evaluating the level of damage instigated by the high temperature on the concrete^4^. Several factors influence the behavior of concrete when exposed to high temperature. These includes the type and properties of the aggregates used, the type and amount of cement, additives and supplementary cementitious materials added, type and amount of fiber added if any^2,5^. To minimize the undesirable effects of elevated temperature on the properties and performance of concrete, it is necessary to be cautious when choosing the basic materials for making the concrete. Evaporation and loss of chemically enclosed water from hydration products of cement reaction in the concrete microstructure initiate when the heat surpasses 110 °C, at this stage the effect of temperature exposure on the concrete’s properties begin to be noticed. As the temperature rises and exceeds 300 °C, dehydration, and thermal expansion of the aggregate’s initiates, causing severe microcracks to form within the matrix of the concrete. As the heat reaches 400 °C, Portlandites disintegration transpires, and when the temperature reaches 600 °C, the disintegration of calcium silicate hydrate (C–S–H) takes places. At about 800 °C, the loss in the hydration products through decomposition becomes highly severe, resulting in substantial loss in compressive strength, ductility, integrity, and durability of the concrete^2,6,7^.

One of the remedies taken to reduce the harmful effect of elevated temperature and improve the concrete’s resistance to high temperature is by adding fibers to the concrete. The fibers are expected to delay the formation and limit the spread of heat induced cracks, and forestalling spalling occurrence in the concrete^3,8^. Studies by Noumowe^9^ disclosed that the addition of 1.8% polypropylene fiber to concrete enhanced its compressive and split tensile strengths as well as its elastic modulus at 200 °C. Another findings by Gencel, Nodehi^10^ observed a gain in compressive strength of concrete when 3% basalt fibers was added and heated at 800 °C. Amin and Tayeh^11^ testified an increase in the flexural and compressive strengths of lightweight concrete with addition of 0.4% polypropylene (PP) and glass fiber. They found that at 400 °C, PP fiber improved the compressive strength by 70%, while glass fiber enhanced the compressive strength by 76%. Talaei and Mostofinejad^3^ reported that the addition of 0.5% steel fiber improved the residual compressive strength of concrete by 20% and 30% at 400 °C and 800 °C respectively.

DPF is a waste material obtained in the form of woven mesh from date trees. The DPF is easy to process for use as a natural fiber in cement composites. When compared to synthetic fibers, the DPF has easier and lower cost of processing, and lower cost to strength ratios^12,13^. Some advantages of using DPF in composites like mortar and concrete includes enhanced ductility and thermal insulation^14,15^. On the contrary, the addition of DPF to concrete and mortar results in significant loss in strengths and durability due to escalation in porosity within the cementitious caused by the DPF’s hydrophilic nature^16–18^. To lessen the damaging effect of the DPF on the properties of concrete and mortar, the DPF is usually treated in alkaline solution. This will make the DPF surface rougher and hence improve the adhesion or bond between the fiber and cementitious matrix. Furthermore, pozzolanic materials like silica fume were added to the DPF to densify its microstructure and reduce the pores produced by the DPF and hence enhance its strength^10^. When DPF is used as a natural fiber in concrete or mortar that will be exposed to high temperature, there is a very high probability that the DPF may degrade easily due to its organic nature and high amount of ligno-cellulose^12,13,19^. Furthermore, as DPF addition to cement composite increase the porosity of the composite microstructure, this will hasten the deterioration of the composite under high temperature, as it will hasten the formation and growth of microcracks due to heat, hence leading to significant decline in strength. Other researchers have also studied the effects of elevated temperature on concrete produced using different natural fibers. Aluko, Yatim^20^ reported that natural fibers containing lignocellulose such as hemp, sisal, coir, and jute fibers can aid in restraining explosive spalling in ultra-high-performance concrete (UHPC) subjected to high temperatures compared to in conventional unreinforced concrete. This is normally achieved through increased porosity and voids in the microstructure of the UHPC by the lignocellulose fibers. Zhang, Tan^21^ investigated the effect of natural fiber (jute) as a replacement to PP fibers in UHPC subjected to high temperature. They observed that the jute fiber swells by absorbing water during mixing and service life and shrinks when exposed to high temperature. This shrinking of the fiber at high temperature creates spaces between voids between the fiber and cement matrix which can have effects on the permeability of the concrete at high temperature. They also observed that with the addition of 3 kg/m^3^ of jute fiber to the UHPC, the compressive strength increased at exposure to 200 °C and reduction in strength at temperature above 400 °C. However, with the addition of 5 kg/m^3^ and 10 kg/m^3^ jute fiber to the UPHC, there was reduction in compressive strength even at room temperature. When the UHPC was subjected to temperature above 400 °C, there was a significant decline in compressive strength. Ozawa, Kim^22^ also reported that the use of jute as natural fiber in high strength concrete (HSC) caused a reduction in compressive strength by up to 40% when heated to a temperature of 100 °C, while at 200 °C and 300 °C, the HSC reinforced with jute fiber recovered its full strength. The jute fiber was able to prevent explosive spalling in the HSC.

Neural networking is one of the best prediction approaches when compared to the traditional methods of statistical forecasting, classification, and recognition^23–25^. The application of neural networks in a variety of human endeavors is a subject that is now receiving a lot of attention. Particularly in the field of construction, use of neural networking is treated as best prediction scheme like in order to semantically segment concrete crack images, Dung^26^ suggested a crack detection system based on deep Fully Convolutional Networks (FCN). The backbone of the FCN encoder, three alternative pre-trained network architectures, were tested for image classification on a public concrete fracture. The average precision achieved by the FCN network is around 90%. The suggested technique for concrete fracture detection was validated using images taken from a film of a cyclic loading test on a concrete specimen. The evaluation of crack density and the reasonable detection of cracks were both shown to be accurate. A deep neural network model was proposed by Nguyen, Kashani^27^ to forecast the compressive strength of foamed concrete. To enhance the performance of the deep neural network model, a new, high-order neuron was created. Moreover, the rectified linear unit activation function and cross-entropy cost function were used to improve the model's performance. Moreover, using a particular data set, the current model was used for predicting the compressive strength of foamed concrete. The findings were then compared with those of other machine learning techniques, such as the Conventional Artificial Neural Network (C-ANN) and Second-Order Artificial Neural Network (SO-ANN). The results showed that density, followed by the water-to-cement and sand-to-cement ratios, had a significant impact on the compressive strength of foamed concrete. The proposed model can assist scientists and engineers in the mixture design optimization of foamed concrete by offering a trustworthy prediction tool. To forecast the compressive strength of rubber concrete, Ly, Nguyen^28^ presented a novel Deep Neural Network model generation process. In order to achieve this, a rubber concrete database was meticulously created, comprising a set of input parameters for the binder, aggregate, and other relevant concrete variables, while the compressive strength was taken into account as an output. A statistical study of the model's prediction outputs is combined with a thorough analysis of the number of hidden layers and the neurons in each layer as part of the creation of the DNN model. The findings demonstrate that, in terms of a number of well-known performance indicators, including coefficient of determination, root mean square error, and mean absolute error, the DNN model outperforms conventional neural network topologies. Also, the proposed DNN model displays higher prediction accuracy than results from earlier publications that used other machine learning algorithms from the literature. The DNN algorithm underwent a sensitivity study utilizing partial dependence plots in order to thoroughly examine the impact of each individual input variable on the anticipated compressive strength of rubber concrete. Using Response Surface Methodology (RSM) and Artificial Neural Networks (ANN) techniques, Kursuncu, Gencel^29^ investigated the effects of Waste Marble Powder (WMP) and Rice Husk Ash (RHA) partially substituting fine aggregate and cement into Foam Concrete (FC) on compressive and flexural strength, porosity, and thermal conductivity coefficient. In the experimental design, the WMP and RHA parameters were determined as three levels, while the foam parameter was calculated as two levels. It was discovered that the ANN technique worked well for estimating the answers. It was discovered that the RSM method worked well for both estimating responses and figuring out the efficient parameters. The recent studies in this regard can be accessed in Refs^30–32^.

Based on the existing study, there are scanty literature that used ANN to predict the properties of silica fume modified DPFRC under high temperature. Hence, in this study ANN was used to predict the residual compressive strength and relative strength of the DPFRC exposed to high temperature condition. Additionally, multivariable regression and Weibull distribution were used for statistical analysis and establishing models for estimating the compressive strengths of the DPFRC concrete under high temperature and carry out reliability analysis of the concrete under elevated temperature.

## Materials and methods

### Materials

**Figure 1:**
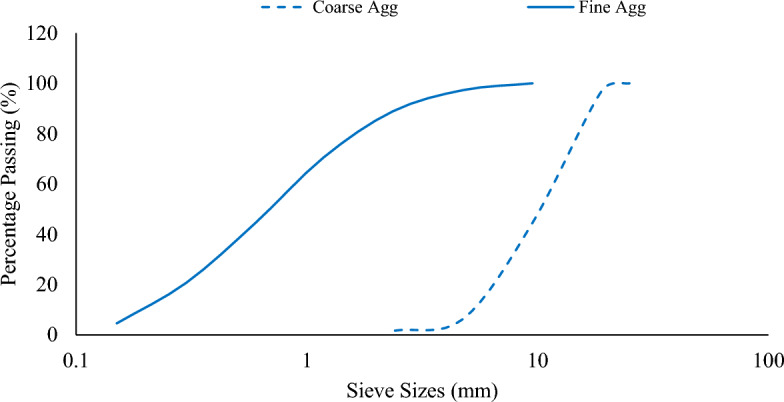
Particle Size distribution of aggregates.

Silica fume and Portland cement were the binder materials employed in this study. The cement conformed with ASTM C150/C150M^33^ specifications. The physical and chemical properties of the binder materials are summarized in Table 1. Natural sand with a maximum size of 4.75 mm in Saturated Surface-Dried (SSD) condition was utilized as the fine aggregate. As revealed in Fig. 1, the fine aggregate has a well graded gradation. Natural crushed gravel with maximum size of 19 mm and in SSD condition was used as the coarse aggregate. Similarly, from Fig. 1, the coarse aggregate was found to be well graded. The physical properties of the aggregates are summarized in Table 2.

**Table 1:** Properties of binders.

Oxides/Properties	Compositions (%)	
	Cement	Silica Fume
Al_2_O_3_	5.390	0.260
CaO	65.180	0.210
Fe_2_O_3_	3.400	0.050
SiO_2_	19.710	95.850
MgO	0.910	0.450
Na_2_O	0.170	–
K_2_O	1.22	–
TiO_2_	0.24	–
SO_3_	3.51	1.00
P_2_O_5_	0.09	–
MnO	0.18	–
LOI	2.38	2.80
SiO_2_ + Al_2_O_3_ + Fe_2_O_3_	28.50%	96.16
Specific Gravity	3.15	2.25
Bulk density (kg/m^3^)	1440	630
Specific Surface Area (m^2^/kg)	325	18,000

**Table 2:** Aggregates properties.

Property	Aggregates	
	Fine	Coarse
Specific gravity	2.63	2.67
Bulk density (kg/m^3^)	1560	1460
Fineness modulus	2.3	–
Water absorption (%)	1.9	0.7

**Figure 2:**
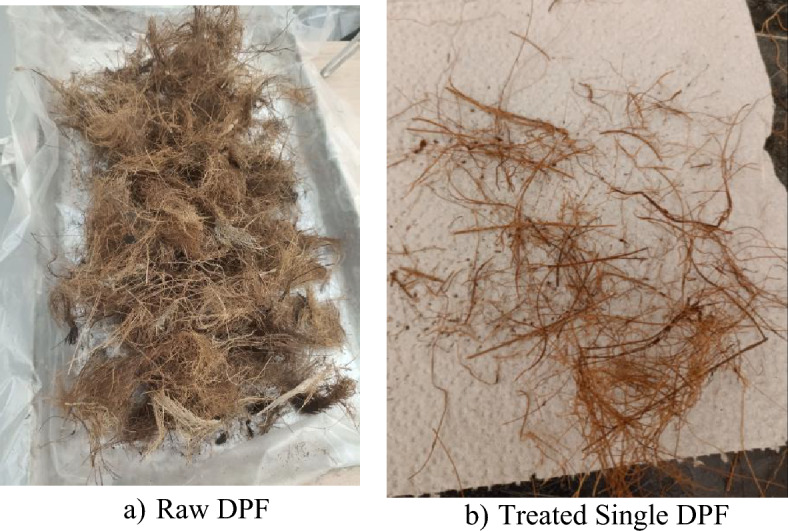
DPF used in this study.

DPF was adopted as a fiber material in this study. The fiber was collected from a neighboring farm in its raw state as shown in Fig. 2a. The raw fiber was obtained in the shape of a quadrilateral mesh of length varying between 300 and 500 mm, and width between 200 to 300 mm. To process the raw fiber for use in concrete, the mesh was fully immersed in clean water for about 2 h, after which it was washed severally with water. An alkaline solution (3% NaOH) was prepared, and the washed fiber was fully immersed in it and kept for 3 h. This treatment was carried out to remove all the excess impurities and dirt from the fiber surface which cannot be removed with normal water and make the fiber surface rougher. After the soaking period, the DPF was cleansed thoroughly using water and then air dried for about 48–72 h until it is completely dried. After drying, the fiber was separated and cut into a single fiber length between 20 and 30 mm and diameters between 0.2 and 1.0 mm as shown in Fig. 2b. This final product of the DPF was used as a natural fiber in concrete.

### Mix Proportioning

The processed DPF was added at varying amounts of 0%, 1%, 2% and 3% by mass of cementitious materials to the concrete. The design of the concrete mix was carried out using the absolute volume method highlighted in ACI 211.1R^34^. Cement was partially replaced with silica fume in the mixes. The dosages of replacements were 0%, 5%, 10% and 15% by volume of cement. To address the effect of variation in water-to-binder ratio in the concrete, a polycarboxylate based water reducing admixture (superplasticizer) was added. The dosage of the admixture was 1% by weight of binder materials in all the mixes. The mixes were produced using the different dosages of silica fume and DPF as presented in Table 3. These mixes were prepared and cast in the laboratory for testing. From Table 3, each of the mixes were given a unique ID based on its content of DPF and silica fume. For illustration, mix M2D0S is the mix containing 2% DPF and 0% silica fume, while mix M3D5S is the mix made with 3% DPF and 5% silica fume, and mix M1D15S is the mix containing 1% DPF and 15% silica fume.

**Table 3:** Mixtures proportions.

Mix	Variable (%)		Quantity (kg/m^3^)						
	DPF (%)	Silica fume (%)	cement	silica Fume	DPF	Fine aggregate	Coarse aggregate	Water	S. P
Control	0.0	0.0	490.0	0.0	0.0	750	905	185	4.9
M1D0S	1.0	0.0	490.0	0.0	4.9	750	905	185	4.9
M2D0S	2	0	490	0.0	9.8	750	905	185	4.9
M3D0S	3	0	490	0.0	14.7	750	905	185	4.9
M1D5S	1	5	465.5	17.9	4.83	750	905	185	4.8
M2D5S	2	5	465.5	17.9	9.67	750	905	185	4.8
M3D5S	3	5	465.5	17.9	14.50	750	905	185	4.8
M1D10S	1	10	441	35.8	4.77	750	905	185	4.8
M2D10S	2	10	441	35.8	9.54	750	905	185	4.8
M3D10S	3	10	441	35.8	14.30	750	905	185	4.8
M1D15S	1	15	416.5	53.7	4.70	750	905	185	4.7
M2D15S	2	15	416.5	53.7	9.40	750	905	185	4.7
M3D15S	3	15	416.5	53.7	14.11	750	905	185	4.7

### Samples preparations

**Figure 3:**
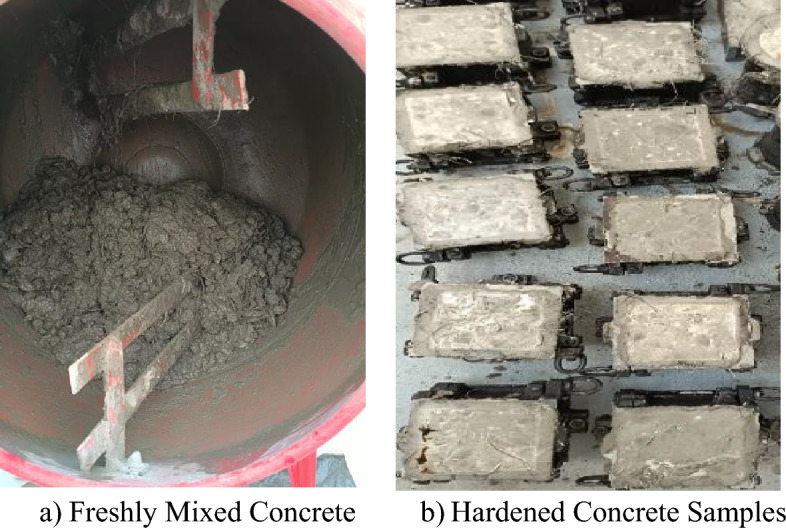
Samples preparation.

The developed mix proportions were produced in the laboratory for testing. Each of the constituent material for the mix under consideration was weighed. The batching, mixing, and casting of the concrete samples were accomplished following the procedures outlined in ASTM C192/C192M^21^. The cement used was also ensured to be free from agglomeration, impurity, and lumps. A rotating drum mixer type available in the laboratory was used for mixing the fresh concrete. The fine aggregate, silica fume and cement were emptied to the mixer, they were then thoroughly mixed for about 45 secs. The DPF and part of the mixing water combined with superplasticizer were added gradually while mixing was going on. The coarse aggregate and remaining part of water combined with superplasticizer were added. The mixing progressed and was stopped after a completely homogenous mix was obtained. The ready mixed concrete in the mixer is shown in Fig. 3a. The freshly mixed concrete was then poured into the 100 mm cube molds which were tight, cleaned, and oiled. The concrete in the cubes were kept in the laboratory for 24 h to settle and hardened. The hardened concrete in the mold is presented in Fig. 3b. After hardening, they were removed from the molds and submerged in clean water for curing.

### Test methods

**Figure 4:**
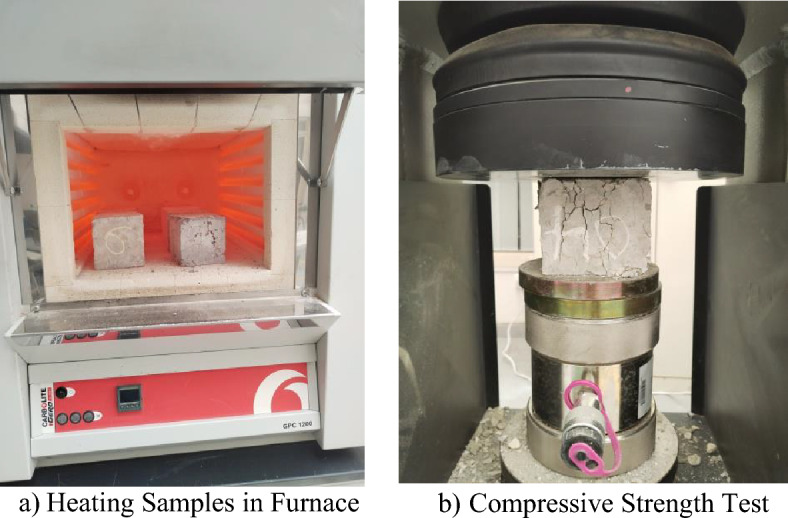
Heating samples inside furnace.

The cube samples were removed from the curing tank after 28 days curing period and air dried for at least 24 h to make them completely dry. The samples were then weighed, and the weight was recorded as W_i_. The samples were subjected to elevated temperature by placing them inside an electric furnace. Each of the mixes was heated at varying temperatures of 24 °C 200 °C, 400 °C, 600 °C and 800 °C for 2 h exposure periods in the electric furnace as shown in Fig. 4a. The heating was done at a fixed rate of 10 °C/min till the chosen temperature is attained, and then allowed to undergo heating at a constant temperature for 2 h. After heat exposure duration is attained, the furnace was turned off and kept closed for the samples to cool completely inside. After cooling, the samples were weighed again, and the weight was recorded as W_F_. Equation (1) was then used to calculate weight loss.

The heated cube samples were then subjected to compressive strength test with the aid of a universal testing machine of 2000 kN capacity following the methods elaborated in BS EN 12,390-3^35^ as shown in Fig. 4b. The relative strengths of each mix were then calculated using Eq. (2).

1 $${W}_{L}\left(\%\right)=\frac{{W}_{i}-{W}_{F}}{{W}_{i}}\times 100$$

2 $${R}_{C}=100-\left[\left(\frac{{F}_{C,i}-{F}_{c,F}}{{F}_{C,i}}\right)\times 100\right]$$

in Eqs. (1) and (2), W_L_ and R_C_ are the weight loss and relative strengths respectively, in %, W_i_ and W_F_ stands for the initial weight prior to heating and final weight after heating respectively, in gs, F_C,i_ and F_C,F_ are the compressive strengths at 24 °C and at the designated heating temperatures respectively in MPa.

## Neural networking model

Use of neural networking models to make predictions is considered by various researchers in different research scenarios^36–39^. Owing to such a directory we offer two different neural networking models to make prediction of Residual Compressive Strength (RCS) and Relative Strength (RS). In detail, neural network is composed of three different layers. Four different inputs, namely date palm fiber, temperature, silica fume, and weight loss are considered in the first layer. The hidden layer owns 10 neurons and case-wise outputs are taken in the last layer. M-I is the case when we take RCS as an output while M-II is the case when we carried RS as an output. The selection of inputs and outputs are offered in Table. 4.

**Table 4:** Parameters for neural model.

Neural model, M-I	Input				Output
	Weight loss (%)	Temperature (°C)	Silica fume (%)	Date palm fiber (%)	Residual compressive strength (Mpa)
Neural model, M-II	Input				Output
	Weight loss (%)	Temperature (°C)	Silica fume (%)	Date palm fiber (%)	Relative strength (%)

**Figure 5:**
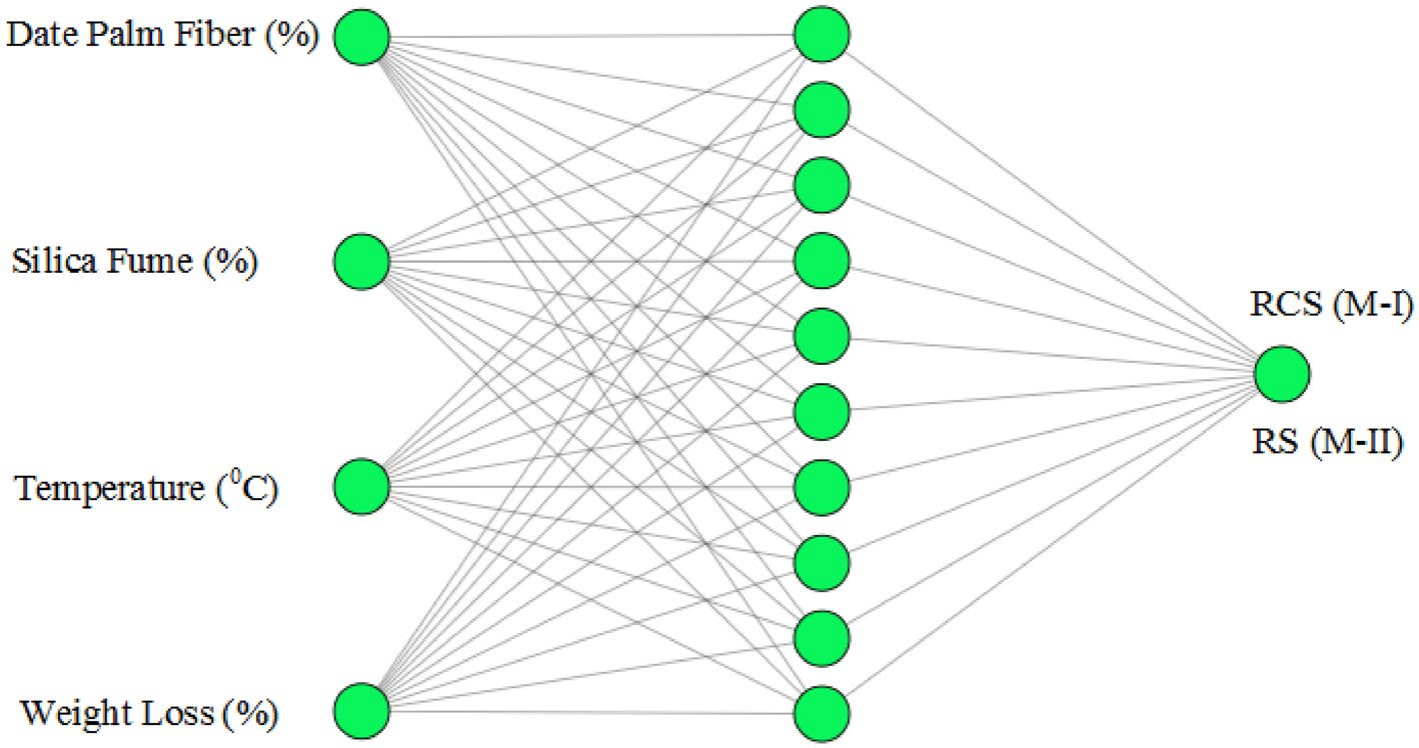
Design of Artificial neural network.

Figure 5 offers the design of artificial neural networking model to predict the Residual Compressive Strength (RCS) and Relative Strength (RS). Training of model is done by using 195 sample of both RCS and RS towards four different inputs. We have considered three samplings namely training, validation, and testing. 137(70%) is slotted for training of neural network while 29(15%) each is used for testing and validation of neural network. The Levenberg–Marquardt backpropagation algorithm (LMBA) is used to training the neural networking models. The following transfer functions given as Eqs. (3) and (4) are used in neural layers:

3 $${F}_{T}\left(x\right)=\frac{1}{1+{e}^{-x}}$$

4 $$Pureline(x)=x$$

Tan-Sig transfer function (Eq. 3) is used in hidden layer while Purelin transfer function (Eq. 4) is used in the output layer. Regression and mean square error relations are mathematically expressed as Eqs. (5) and (6) as follows:

5 $$R=\sqrt{1-\frac{\sum_{j=1}^{n}{({X}_{num\left(j\right)}-{X}_{ANN\left(j\right)})}^{2}}{\sum_{j=1}^{n}{({X}_{num\left(j\right)})}^{2}}}$$

6 $$MSE=\frac{1}{n}\sum_{j=1}^{n}{({X}_{num\left(j\right)}-{X}_{ANN\left(j\right)})}^{2}$$

## Results and discussion

### Experimental results

The results of the weight loss, residual compressive strength, and relative strengths of the DPF reinforced concrete containing silica fume as supplementary cementitious material presented in Table 5 was based on the previous studies by Adamu, Ibrahim^40^, and has been reported in this study to enhance readability and ease of understanding the findings in this study based on the ANN, Weibull distribution and multivariable regression analysis models developed. The weight loss of the DPFRC intensified with rise in temperature which was due to the loss of capillary water and gels from the microstructure of the concrete. At higher temperatures of 600 °C and above, the weight loss was due to evaporation of chemically bound water from the hydration products. Furthermore, there was an escalation in weight loss with increment in percentage addition of DPF to the concrete. This was because DPF creates more voids in the concrete, therefore under high temperature capillary water and chemically bound water evaporates easily from the voids, thereby escalating the weight loss in the DPFRC. The partial substitution of cement with silica fume in the DPFRC decreased the weight loss when heated up to a temperature of 400 °C. This is due to the pozzolanic reaction between the silica fume and cement hydration products which densified the concrete microstructure and prevent the evaporation of water from the concrete under high temperature^40,41^. The strengths of the DPFRC improved when subjected to elevated temperature of up to 400 °C regardless of the proportions of DPF and silica fume in the mixes. This improvement in strength can be linked to the formation of excess tobermorite from the hydrothermal reactions between the SiO_2_ from silica fume and calcium oxide from cement. The addition of DPF to the concrete resulted in reduction in residual and relative strengths of the concrete due to premature failure occurring in the concrete due to voids created by the DPF in the microstructure of the concrete. Additionally, the poor adhesion between the DPF and cement paste also caused a reduction in strengths at any temperature. The strengths of the DPFRC improved with partial replacement of not more than 10% cement with silica fume at a temperature of up to 600 °C. The improvement in strength might be due to the consumption of excess Portlandites (Ca(OH)_2_) thereby inhibiting its conversion to lime at high temperature, hence avoiding the damage that can be caused by this reaction^40,41^.

**Table 5:** Results of weight loss and strengths of DPFRC under high temperature^40^.

Mix/Temp	Weight Loss				Residual strength (MPa)					Relative strength (MPa)			
	200 °C	400 °C	600 °C	800 °C	24°C	200 °C	400 °C	600 °C	800 °C	200 °C	400 °C	600 °C	800 °C
Control	0.59	0.94	3.52	4.47	43.91	45.26	50.90	31.44	23.38	103.1	115.9	71.6	53.2
M1D0S	0.63	1.14	4.71	6.91	42.04	44.11	47.62	21.66	16.08	104.9	113.3	51.5	38.2
M2D0S	0.75	1.42	5.51	7.87	34.61	39.17	40.07	20.69	14.88	113.2	115.8	59.8	43.0
M3D0S	0.96	1.77	6.92	9.51	35.02	33.29	20.22	14.79	10.95	95.1	57.7	42.2	31.3
M1D5S	0.51	0.85	5.44	7.97	46.22	49.24	54.03	26.39	17.96	106.5	116.9	57.1	38.9
M2D5S	0.55	0.91	6.15	8.44	51.17	56.45	59.11	30.07	13.17	110.3	115.5	58.8	25.7
M3D5S	0.67	1.46	7.20	9.93	36.00	43.96	40.83	21.51	10.11	122.1	113.4	59.8	28.1
M1D10S	0.32	0.84	5.97	8.20	54.12	58.12	66.69	27.72	14.15	107.4	123.2	51.2	26.1
M2D10S	0.43	0.81	6.83	8.58	49.80	54.98	57.43	21.47	12.91	110.4	120.7	43.1	25.9
M3D10S	0.58	1.13	7.56	10.25	37.25	46.27	36.50	19.12	10.54	124.2	98.0	51.3	28.3
M1D15S	0.48	0.75	6.79	8.79	33.42	47.01	52.92	14.18	8.41	140.6	158.3	42.4	25.2
M2D15S	0.69	1.11	7.01	9.78	28.74	35.20	45.52	15.08	7.34	122.5	158.4	52.5	25.5
M3D15S	0.76	1.68	7.29	10.46	26.56	33.97	36.46	14.02	8.91	127.9	137.3	52.8	33.5

### Neural networking analysis

**Figure 6:**
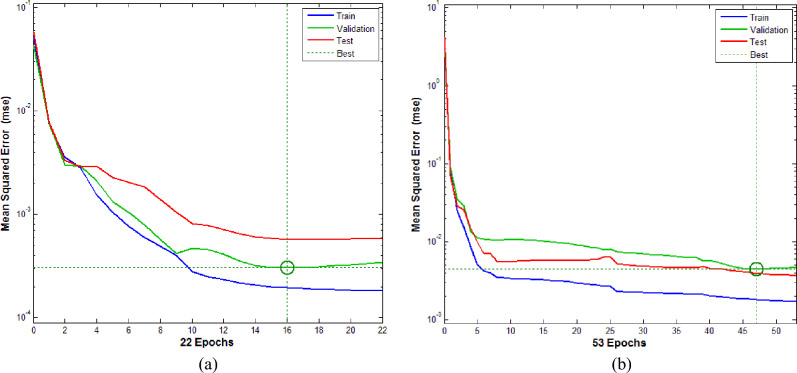
(**a**) Training performance outcomes for M-I. (**b**) Training performance outcomes for M-II.

**Figure. 7:**
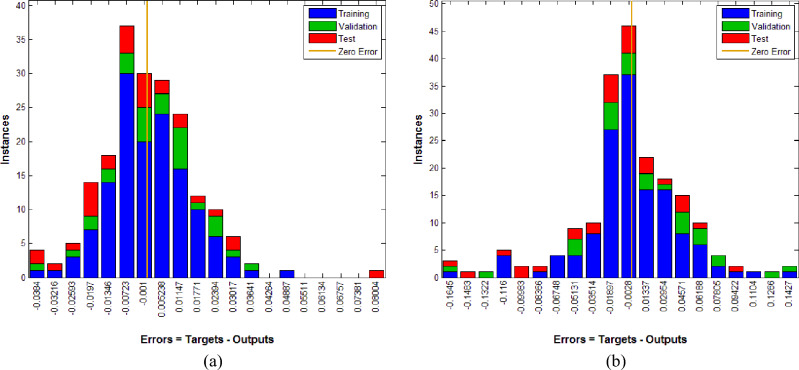
(**a**) Error histogram outcome for M-I. (**b**) Error histogram outcome for M-II.

Both RCS and RS are predicted by using ANN models namely M-I and M-II respectively. Figures 6a, 7b are the ultimate training performance outcomes for ANN models. In detail, Fig. 6a gives performance pattern for neural networking to predict the RCS and Fig. 6b gives the performance pattern for neural networking to predict the RS.

From Fig. 6a one can see that the mean square error is high at the initial stage while for upcoming epochs the error reduces significantly. We have seen that the best validation performance for the model M-I is 0.000305017 at epoch 16.

From Fig. 6b, one can see that for the first 4 epochs the mean square error is high. For higher epochs the error significantly reduces to desired values. The best validation performance for the model M-II is 0.0044732 at epoch 47. Figure 7a gives the error histogram for ANN model to predict RCS and Fig. 7b gives the error histogram for ANN model to predict RS. One can see that the training of neural working model is finished reasonably.

**Figure 8:**
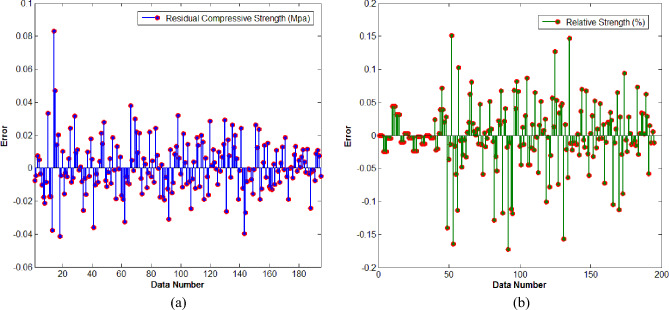
(**a**) Error bar outcome for M-I. (**b**) Error bar outcome for M-II.

**Figure 9:**
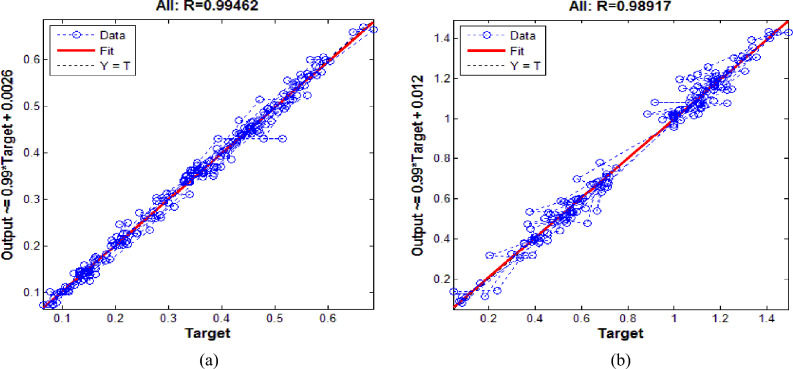
(**a**) Regression outcome for M-I. (**b**) Regression outcome for M-II.

**Figure 10:**
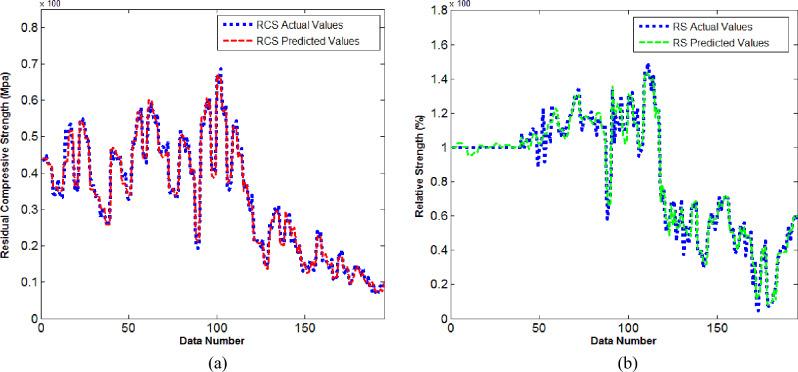
(**a**) Comparative outcome for M-I. (**b**) Comparative outcome for M-II.

Figure 8a offers the error bar for the predictive values of RCS with targeted values of RCS while Fig. 8b gives the error bar for the predictive values of RS with its targeted values. In detail, from Fig. 8a one can see that the error values from mean line are not far away. Most of the predictive values carry errors in between − 0.02 to + 0.02 which seems acceptable. Some values carry errors within the band range of − 0.04 to + 0.04. Out of 195 sample values only four predictive values carry error within range of − 0.08 to + 0.08. In Fig. 8b, we can see that maximum predictive values carry error within bandwidth of − 0.05 to + 0.05. Out of 195 samples values only 05 values carry error in between − 0.15 to + 0.15. The regression plots for both neural networking models are offered in Fig. 9a,b. In detail, Fig. 9a offers the regression outcome for ANN model constructed to predict the RCS while Fig. 9b gives the regression outcome for ANN model constructed to predict the RS. It is important to note that in Fig. 9a,b, All: refers to collective value of regression for training, validation and testing. We have seen that for predictive model M-I, the regression is noticed R = 0.99462. The value is close enough to R = 1 which implies the strong correlation of predictive and targeted values of RCS. Further, the regression value R = 0.98917 is noticed for predictive model M-II. Such value is collective in response to training, validation, and testing. Owing to such value we can say that the predictive values of RC by using ANN are closely related to experimental values of RS. Figure 10a offers the comparative values of experimental values of Residual Compressive Strength with predictive values of Residual Compressive Strength by using artificial neural networking model. We have noticed that predicted values of RCS are matchable with actual values. Figure 10b offers the comparison of predicted values of Relative Strength with actual values of Relative Strength. One can see that predicted and actual values are in agreement. Therefore, the constructed neural models M-I and M-II have capacity to predict values of RCS and RS with high accuracy.

Tables 6 and 7 offers the characteristics of developed neural networking model. In detail, the characteristics of neural networking model M-I is offered in Table 6 while Table 7 gives the characteristics of neural networking model, M-II. For both M-I and M-II, we have seen that the mean square values are appreciable low which leads to accuracy of constructed neural networking model. Further, the regression values admit the strong correlation of targeted values and predicted values of RCS and RS by neural networking models M-I and M-II respectively.

**Table 6:** Characteristics of neural networking model, M-I.

Grades	Training	Validation	Testing
MSE	1.9583e^−04^	3.0517e^−04^	5.7403e^−04^
Regression	0.9964	0.9923	0.9850

**Table 7:** Characteristics of neural networking model, M-II.

Grades	Training	Validation	Testing
MSE	0.0018	0.0045	0.0039
Regression	0.9921	0.9863	0.9829

### Statistical modelling using multivariable regression

A statistical modelling using multivariable regression analysis (MRA) was carried out to establish model equations for calculating or forecasting the properties of the DPFRC exposed to elevated temperature using DPF, silica fume and temperature as the inputs.

The basic globalized MRA equation is expressed in form of linear equation expressed as Eq. (7)^42^.

7 $$y\left({x}_{1},{x}_{2},{x}_{3}\dots \dots {x}_{n}\right)={\psi +{\rm Z}}_{1}{x}_{1}+{\rm Z}_{2}{x}_{2}+{\rm Z}_{3}{x}_{3}+\dots {\rm Z}_{n}{x}_{n}$$

For which y denotes the response/output, Ψ is the coefficient of the intercept, Ζ is the linear regression coefficients.

However, as most of the relationships between the responses/outputs and variables contains some curvature, non-linear multi-variable regression analysis using second-order polynomial will improve the prediction accuracy and reduce the errors in the models. The global second-order non-linear MRA equation is expressed as Eq. (8)^42^.

8 $$y={\sum }_{i=1}^{n}{\rm Z}_{i}{x}_{i}+{\sum }_{i=1}^{n-1}{\sum }_{j=i+1}^{n}{\rm Z}_{ij}{x}_{i}{x}_{j}+\psi $$

where y stands for the response/output, x stands for the independent variables, Ζ_i_ represents the coefficient of the 1st order polynomial in the equations, and Ζ_ij_ represents the coefficient of the 2nd order polynomial in the equation, n is the number of input variables, and Ψ is the constant of regression.

In this research, the non-linear MRA was employed to develop models for forecasting and calculating the strengths and weight loss for the DPFRC concrete exposed to high temperature.

#### ANOVA for developed models

The developed mathematical equations for the prediction of the weight loss, residual compressive strength and relative strength of the DPFRC containing silica fume under elevated temperature is given as Eqs. (9)–(11) respectively.

9 $${W}_{L}=0.00065*D+0.0639*S+0.0144*T+0.0124*D*S+0.00019*D*T+4.6\times {10}^{-5}*S*T-0.0178*{D}^{2}+0.00139*{S}^{2}-6.59\times {10}^{-6}*{T}^{2}-0.974$$

10 $${F}_{C}=-2.34*D+2.5475*S+0.0414*T+0.13*D*S+0.00164*D*T-0.000425*S*T-1.175*{D}^{2}-0.1834*{S}^{2}-9.93\times {10}^{-5}*{T}^{2}+45.07$$

11 $${R}_{C}=-4.64*D-1.666*S+0.096*T+0.328*D*S+0.0038*D*T-0.00098*S*T+0.117*{D}^{2}+0.141*{S}^{2}-0.00023*{T}^{2}+104.56$$

In the above Eqs, W_L_ represents the weight loss in %, Fc and RC are the residual and relative compressive strengths respectively, in MPA and % respectively. T is temperature in ^°^C, D is DPF in %, and S is silica fume in %.

Table 8 highlights the ANOVA for all the models established for the estimation of the DPFRC’s properties under high temperature. Using the level of significance (*P* < 0.05), the statistical significance for all the models were checked. All the models were found to be statistically significant based on the significance test (*P* < 0.05), as their P-values are less than 0.05. Hence the statistical null hypotheses for all the models were false and rejected. In terms of R^2^, higher values of R^2^ clarified the high degree of correlation of the models with the experimental results and hence high predicting accuracy. From Table 8, the weight loss model has an excellent R^2^ value of 0.928 which is very close to 1, and hence the model is said to have a very high accuracy and prediction. For the residual and relative strength models, their R^2^ values of 0.818 and 0.786 are said to be very good and also close to 1, and hence the models have good correlation and prediction ability. all models can be said to have excellent R^2^ scores which is ≥ 0.9. Further model accuracy test was done by comparing the standard errors (SE) for each model relative to its mean square values. The low SE relative to mean square for each of the model explains the accuracy of prediction with less errors.

**Table 8:** ANOVA summary for multivariable regression models.

Output/model	df		SS		MS		SE	F-value	*P*-value	R^2^
	Reg	Res	Reg	Res	Reg	Res				
Weight loss	9	55	508.418	39.229	56.491	0.713	0.845	79.201	3.14 × 10^−28^	0.928
Residual compressive Strength	9	55	13,199.7	2940.5	1466.64	53.464	7.312	27.432	2.89 × 10^−17^	0.818
Residual Strength	9	55	58,297.64	15,845.5	6477.52	288.1	16.97	22.484	2.04 × 10^−15^	0.786

*df = degrees of freedom, Reg = Regression, Res = Residual, SS = Sum of Squares, MS = Mean Squares, SE = Standard Errors.

#### Diagnostic plots

**Figure 11:**
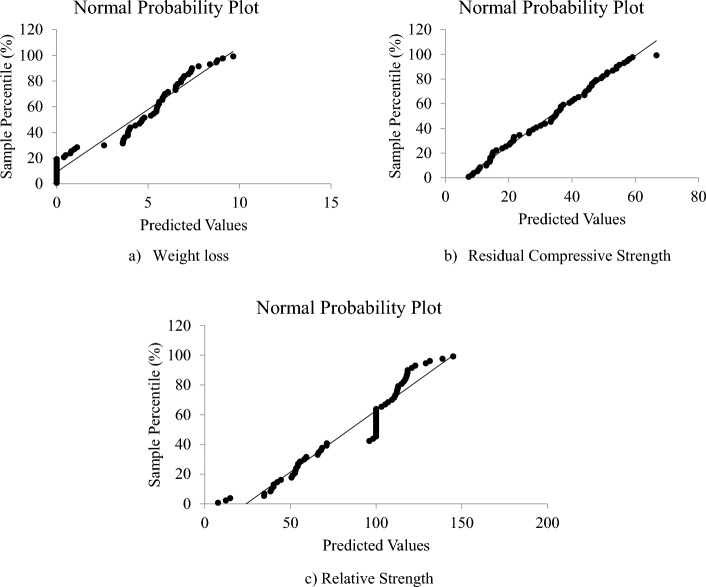
Normal probability plots.

The normal probability graphs for the weight loss, residual compressive strength and residual strength models are presented as Fig. 11a–c, respectively. All the models a reasonably good correlation with the straight trend line drawn on its plot. Therefore, the experimental data and the models for the DPFRC containing silica fume under high temperature all followed and agreed with the normal probability distribution. By comparing the normal plots of the models, it can be clearly seen that the residual compressive strength model (Fig. 11b) has the best fit with its straight trend line, and hence is said to be the most normally distributed model.

**Figure 12:**
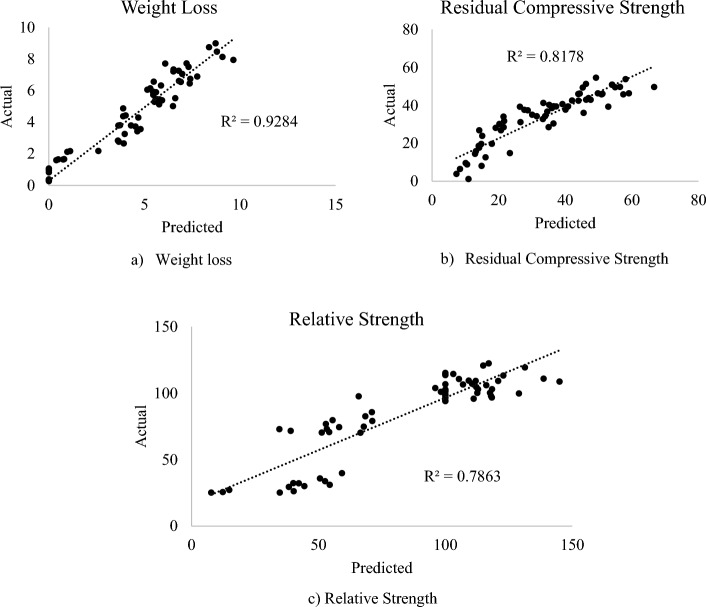
Predicted versus actual plots.

Figure 12 highlights the predicted values against the experimental data graphs for the DPFRC models modified with silica fume exposed to elevated temperature. The weight loss model has the highest fitness between its predicted and actual values with R^2^ of 0.93 as seen in Fig. 12a. The relative strength model has the least fitness between its predicted and actual values when compared to the other models, with an R^2^ of 0.79. From the plots only about 7%, 18% and 21% of the total data for the weight loss, residual compressive strength and relative strength models respectively were not perfectly fitted with the developed mathematical models. Consequently, the proposed models for predicting or forecasting the weight loss and strengths of the DPFRC containing silica fume under high temperature can be said to have a reasonably good to high prediction accuracy.

### Statistical analysis using Weibull distribution.

#### Weibull distribution function

Weibull distribution is a numerical approach that is normally used by engineers for the analysis of brittle materials failure. Several research outcomes reveal that the Weibull distribution parameters are linked with the sizes and types of flaws^43^. In addition, Weibull statistical analysis can also be used to analyse quasi-brittle materials^44^. The fracture toughness and strengths of quasi brittle materials like rocks followed the Weibull distribution., where the two and three parameters Weibull distribution functions were utilized for analysing the rock fracture’s probability^44,45^. Concrete is considered as a quasi-brittle composite material due to variation in its strength and other mechanical properties which are dependent on the type and quantity of cementitious materials, aggregate type and strength, temperature and humidity, casting and curing methods. Therefore, concrete’s strength and other properties can be analysed using Weibull distribution^43^. Previous research has adopted Weibull distribution for the analysis of concrete’s properties. He and Xie^46^ analysed the compressive and tensile strengths of concrete using Weibull distribution and reported a very high correlation for the models where the concrete strengths obeyed the two-parameter Weibull distribution function. Kencanawati, Iizasa^47^ used Weibull distribution for the analysis of the strengths and reliability of recycled aggregate concrete (RAC). They found a good correlation between the Weibull analysis and mechanical strengths of the concrete and concluded that the Weibull distribution can be used for reliability analysis of the RAC. The dynamic and static mechanical properties of FRC have been evaluated using Weibull distribution statistical method and was reported to have a high prediction and correlation^48,49^. The drop weight impact resistance test on concrete was analysed statistically using Weibull distribution functions^48,50^. The Weibull distribution was found to be a suitable statistical analysis method for impact and fatigue tests on concrete due to its nature of enhancing hazard function, which characterize the real behaviour of engineering constructions under repeated loads^51^. Zheng, Cheng^52^ modified the Weibull distribution to a simplified form with lesser parameters and containing the pre-peak and post-peak stresses in shotcrete. They found that that the modified Weibull distribution model effectively quantified the failure mechanism of the shotcrete, and the Weibull model was reasonably in good correlation with the experimental data, with the model having an acceptable reliability. Guo, Qiao^53^ used the two and three parameters Weibull distributions to carryout stochastic analysis for the assessment of concrete structure’s durability. They found that the Weibull distribution is well fitted for the analysis of the life data and durability of the concrete structures throughout its service life. The three parameters Weibull distribution model has the best fit compared to the two parameters Weibull distribution. Shahraki, Hua^54^ employed Weibull distribution to establish probabilistic models for predicting the residual compressive strengths of calcareous and siliceous concrete when exposed to temperature between 200 to 1000 °C for 2 h exposure. They defined the parameters of the Weibull distribution as a function of temperature in continuous closed-form solutions. Their findings showed that the model for the residual compressive strength followed the Weibull distribution, and the model can be employed for computational and analytical frameworks.

The cumulative probability function of the Weibull distribution W(N) is used for the estimation and prediction of the residual compressive strength and relative strengths of the DPFRC subjected to elevated temperature as presented in Eq. (12) below^55^.

12 $$Y\left(W\right)=\frac{\gamma }{\mathrm{\rm T}-{\mathrm{\rm Z}}_{0}}{\left[\frac{\mathrm{\rm Z}-{\mathrm{\rm Z}}_{0}}{\mathrm{\rm T}-{\mathrm{\rm Z}}_{0}}\right]}^{\gamma -1}*Exp\left\{-{\left[\frac{\mathrm{\rm Z}-{\mathrm{\rm Z}}_{0}}{\mathrm{\rm T}-{\mathrm{\rm Z}}_{0}}\right]}^{\gamma }\right\}\left(\mathrm{\rm T}\le \mathrm{\rm Z}\le \infty \right)$$

By integrating Eq. (12), the random variable distribution function F(W_P_) is derived as presented in Eq. (13).

13 $$Y\left({W}_{P}\right)=P\left(W\le {W}_{P}\right)=1-Exp\left\{-{\left[\frac{\mathrm{\rm Z}-{\mathrm{\rm Z}}_{0}}{\mathrm{\rm T}-{\mathrm{\rm Z}}_{0}}\right]}^{\gamma }\right\}$$

In Eqs. (12) and (13), γ and Ζ denotes the shape parameter and characteristic life (scale factor) respectively, for the Weibull distribution, T represents the definite value of the random variable W, Ζ_0_ is the parameter of the location for which Ζ ≥ Ζ_0 and T_ ≥ Ζ_0._

Supposing the minimum concrete life Ζ_0_ = 0 for the compressive strengths of the DPFRC under high temperature, then the survival probability function F(S_P_) can be presented as Eq. (14).

14 $$F\left({S}_{P}\right)=1-Exp\left\{-{\left[\frac{\mathrm{\rm Z}}{\mathrm{\rm T}}\right]}^{\gamma }\right\}$$

Now simplifying Eq. (14) by taking natural log of both sides twice returned Eq. (15)

15 $$Ln\left\{Ln\left[\frac{1}{F({S}_{P})}\right]\right\}=\gamma Ln\left(\mathrm{\rm Z}\right)-\gamma Ln(\mathrm{\rm T})$$

Lastly, Eq. (15) can be utilized for checking if the compressive strengths of the DFPRC mixes exposed to high temperature followed the two-parameter Weibull distribution function, which is carried out by solving the Eq. (15) above and then relating it with the normal straight-line equation (Y = Mx + C) to get γ, γLn(Ζ) and γLn(T). From the expression the degree of correlation (R^2^) of the line can also be obtained and used to explain the accuracy of the Weibull distribution^50^.

Equation (16) depicts the survival probability function.

16 $$P\left({S}_{P}\right)={P}_{J}=1-\frac{M}{N+1}$$

In the above Eq. (16), M represents the strength loss order number of the residual or relative compressive strength after subjecting to high temperature, rearranged in rising order, N represents the number of mixes of the DFPRC.

Due to the fact that subjecting concrete to elevated temperature will increase its quasi-brittle nature and causes further variation in its strength, this study utilized the Weibull distribution function for the probabilistic modelling and reliability analysis of DPFRC containing silica fume under elevated temperatures.

#### Analysis of compressive strengths of DPFRC using Weibull distribution

**Figure 13:**
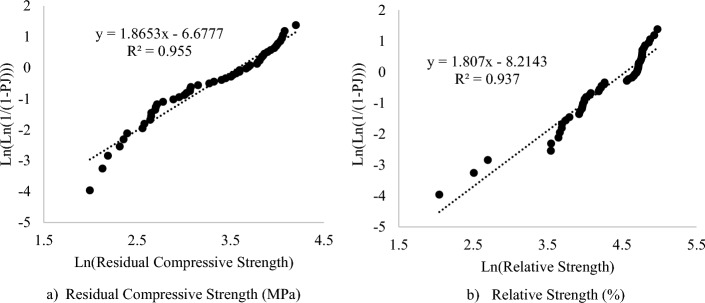
Weibull distribution plots.

The residual compressive strength (Fc) and relative strengths (Rc) of the DPFRC modified with silica fume exposed to elevated temperatures between 200 and 800 °C was analysed using Weibull distribution. The Weibull distribution parameters is presented in Table 9 and is used to plots the Weibull plots and find the coefficient of determination (R^2^). The plots of Ln(Ln(1/P_J_) against Ln(Fc) is presented as Fig. 13a, while the graph of Ln(Ln(1/P_J_) against Ln(Rc) is given as Fig. 13b. The ANOVA for the Weibull distribution analysis is given in Table 10. From Fig. 13a,b, the Weibull charts for all the strengths demonstrates and followed a linear pattern with a very high R^2^s superior to 0.93. Hence, the residual compressive strength and relative strength of the DFPRC when exposed to elevated temperature followed the two-parameter Weibull distribution^56,57^. Hence the Weibull distribution function can be used for evaluation of the reliability or failure probability of the DPFRC under high temperatures. Shahraki, Hua^54^ have also found a similar result, where they reported the residual compressive strength of calcareous and siliceous concrete at temperature up to 1000 °C followed the two-parameter Weibull distribution function.

**Table 9:** Weibull distribution parameters.

Residual Strength (Fc) (MPa)			Relative Strength (Rs) (%)			Weibull Distribution Parameters			
Mixes	Fc (MPa)	Ln(Fc)	Mixes	Rs (%)	Ln(Rs)	Rank	P_J_	Ln(1/P_J_)	Ln(Ln(1/P_J_))
M1D5S-400	66.69	4.200	M1D10S-400	145.00	4.977	52	0.019	3.970	1.379
M2D5S-400	59.11	4.079	M2D15S-400	138.73	4.933	51	0.038	3.277	1.187
M1D5S-200	58.12	4.063	M1D10S-200	131.36	4.878	50	0.057	2.872	1.055
M2D10S-400	57.43	4.051	M1D5S-400	129.02	4.860	49	0.075	2.584	0.949
M2D5S-200	56.45	4.033	M3D15S-400	122.85	4.811	48	0.094	2.361	0.859
M2D10S-200	54.98	4.007	M3D10S-200	120.81	4.794	47	0.113	2.179	0.779
M1D5S-400	54.03	3.990	M3D5S-200	118.38	4.774	46	0.132	2.024	0.705
M1D10S-400	52.92	3.969	M2D5S-400	118.34	4.774	45	0.151	1.891	0.637
M0D0S-400	50.90	3.930	M1D5S-400	118.02	4.771	44	0.170	1.773	0.573
M1D5S-200	49.24	3.897	M2D10S-400	117.61	4.767	43	0.189	1.668	0.511
M1D0S-400	47.62	3.863	M3D15S-200	117.10	4.763	42	0.208	1.572	0.453
M1D10S-200	47.01	3.850	M0D0S-400	116.14	4.755	41	0.226	1.485	0.396
M3D10S-200	46.27	3.834	M2D15S-200	114.91	4.744	40	0.245	1.405	0.340
M2D15S-400	45.52	3.818	M1D0S-400	112.88	4.726	39	0.264	1.331	0.286
M0D0S-200	45.26	3.812	M2D0S-400	112.61	4.724	38	0.283	1.262	0.233
M1D0S-200	44.36	3.792	M2D5S-200	112.20	4.720	37	0.302	1.198	0.180
M3D5S-200	43.96	3.783	M2D10S-200	111.96	4.718	36	0.321	1.137	0.128
M3D5S-400	40.83	3.709	M3D5S-400	111.16	4.711	35	0.340	1.080	0.077
M2D0S-400	40.07	3.691	M2D0S-200	110.53	4.705	34	0.358	1.026	0.026
M2D0S-200	39.17	3.668	M1D5S-200	109.23	4.693	33	0.377	0.975	− 0.026
M3D10S-400	36.50	3.597	M1D5S-200	106.96	4.672	32	0.396	0.926	− 0.077
M3D15S-400	36.46	3.596	M1D0S-200	105.36	4.657	31	0.415	0.879	− 0.129
M2D15S-200	35.20	3.561	M0D0S-200	103.12	4.636	30	0.434	0.835	− 0.181
M3D15S-200	33.97	3.525	M3D10S-400	98.26	4.588	29	0.453	0.792	− 0.233
M3D0S-200	33.29	3.505	M3D0S-200	96.01	4.564	28	0.472	0.751	− 0.286
M0D0S-600	31.44	3.448	M0D0S-600	71.22	4.266	27	0.491	0.712	− 0.339
M2D5S-600	30.07	3.404	M3D15S-600	71.04	4.263	26	0.509	0.674	− 0.394
M1D5S-600	27.72	3.322	M2D15S-600	68.47	4.226	25	0.528	0.638	− 0.449
M1D5S-600	26.39	3.273	M2D0S-600	67.87	4.218	24	0.547	0.603	− 0.506
M0D0S-800	23.38	3.152	M3D5S-600	66.56	4.198	23	0.566	0.569	− 0.564
M1D0S-600	21.65	3.075	M3D0S-400	65.84	4.187	22	0.585	0.536	− 0.623
M3D5S-600	21.51	3.069	M3D15S-800	59.25	4.082	21	0.604	0.505	− 0.684
M2D10S-600	21.47	3.067	M3D10S-600	58.14	4.063	20	0.623	0.474	− 0.747
M2D0S-600	20.69	3.030	M1D10S-600	55.58	4.018	19	0.642	0.444	− 0.812
M3D0S-400	20.22	3.007	M2D0S-800	54.46	3.998	18	0.660	0.415	− 0.880
M3D10S-600	19.12	2.951	M1D5S-600	54.22	3.993	17	0.679	0.387	− 0.950
M1D5S-800	17.96	2.888	M3D0S-600	53.31	3.976	16	0.698	0.359	− 1.023
M1D0S-800	16.08	2.778	M1D0S-600	52.94	3.969	15	0.717	0.333	− 1.100
M2D15S-600	15.08	2.713	M0D0S-800	52.62	3.963	14	0.736	0.307	− 1.182
M2D0S-800	14.88	2.700	M2D5S-600	51.30	3.938	13	0.755	0.281	− 1.268
M3D0S-600	14.79	2.694	M2D15S-800	50.60	3.924	12	0.774	0.257	− 1.360
M1D10S-600	14.18	2.652	M3D0S-800	44.44	3.794	11	0.792	0.233	− 1.458
M1D5S-800	14.15	2.650	M1D10S-800	42.26	3.744	10	0.811	0.209	− 1.565
M3D15S-600	14.02	2.640	M3D5S-800	40.24	3.695	9	0.830	0.186	− 1.681
M2D5S-800	13.17	2.578	M1D0S-800	40.07	3.691	8	0.849	0.164	− 1.810
M2D10S-800	12.91	2.558	M1D5S-600	39.06	3.665	7	0.868	0.142	− 1.954
M3D0S-800	10.95	2.393	M3D10S-800	38.33	3.646	6	0.887	0.120	− 2.119
M3D10S-800	10.54	2.355	M1D5S-800	34.76	3.548	5	0.906	0.099	− 2.312
M3D5S-800	10.11	2.314	M2D10S-600	34.60	3.544	4	0.925	0.078	− 2.545
M3D15S-800	8.91	2.187	M2D10S-800	14.84	2.698	3	0.943	0.058	− 2.843
M1D10S-800	8.41	2.129	M2D5S-800	12.30	2.509	2	0.962	0.038	− 3.258
M2D15S-800	7.34	1.993	M1D5S-800	7.73	2.046	1	0.981	0.019	− 3.961

**Table 10:** ANOVA for regression coefficients of Weibull distributions.

Properties at elevated temperature	α (shape factor)	Regression coefficient αLn(u)	Characteristic life/scale factor (u)	R^2^
Residual compressive strength (MPa)	1.8653	6.678	35.872	0.955
Relative strength (%)	1.807	8.214	94.238	0.937

#### The Anderson–darling test hypotheses

The Anderson–Darling test (AD-value) and p-value were used to test if the residual compressive strength and residual strengths followed the normal probability distribution. The AD statistical values was calculated based on the following equations^58–60^.

17 $$S=(2i-1)\left[lnF\left({X}_{i}\right)+\mathrm{ln}(1-F\left({X}_{n-i+1}\right))\right]$$

18 $$AD=-n-\frac{1}{n}\sum_{i=1}^{n}{S}_{i}=0.288$$

19 $${AD}^{*}=AD\left(1+\frac{0.75}{n}+\frac{2.25}{{n}^{2}}\right)$$

where n = sample size, F(X) = cumulative distribution function for the specified distribution and i = the i^th^ sample when the data is sorted in ascending order

**Figure 14:**
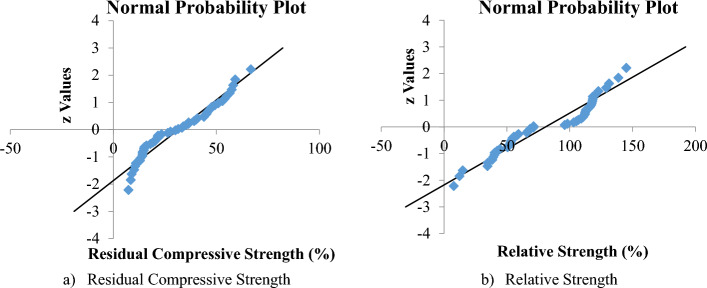
The Anderson–Darling test plots.

The AD statistical values for the residual and relative strengths were plotted as shown in Fig. 14a,b respectively. The Probability (P-values) were then calculated using the formulas obtained from R.B. D’Augostino and M.A. Stephens, Eds., 1986^61^ and summarized in Table 11. From Table 11, the *P* values for both the residual compressive strength and relative strength are very low (less than 0.05), hence the *P*-values of the AD statistic is not significant, hence the data does not follow the normal distribution. Therefore, Weibull distribution analysis which was used for the statistical analysis of the residual compressive strength and relative strength of the DPFRC subjected to high temperature is more appropriate.

**Table 11:** P-values obtained from AD test.

Statistical terms	Residual compressive strength (MPa)	Relative strength (%)
Sample sizes (n)	52	52
S	− 2764.13	− 2797.61
Anderson–Darling (AD)-value	1.156276	1.800283
Adjusted AD-value (AD*)	1.173916	1.827747
Probability Value (p-value)	0.004596	0.000114

#### Reliability analysis

The compressive strengths of the DPFRC under high temperature followed the two-parameter Weibull distribution as obtained in Sect. 3.2.3. Hence Weibull analysis was implemented for the estimation of the residual compressive strength and relative strengths of the DPFRC which corresponds to different survival probability (reliability). Equation (17) is used to estimate the different failure probabilities which correspond to the residual compressive strengths and relative strengths of the DPFRC.

20 $${F}_{C}={R}_{C}=\mathrm{\rm Z}{\left\{Ln(1-{P}_{f}\right\}}^{\frac{1}{\gamma }}$$

In the above equation, Fc and Rc represents the residual compressive strength and relative strength respectively, P_f_ represents probability of failure, Ζ and γ denotes the scale factor and shape factor respectively^57^.

Finally, the survival probability or design reliability (S_P_) is obtained using Eq. (18)

21 $${S}_{P}=1-{P}_{f}$$

**Figure 15:**
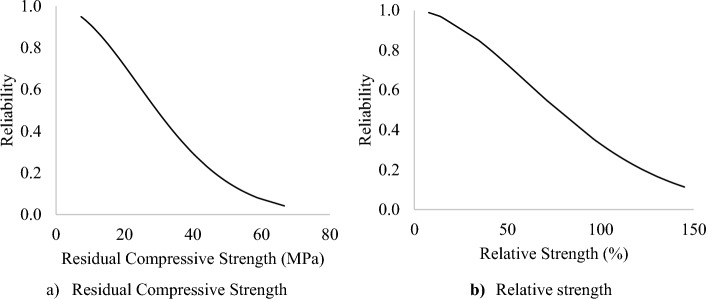
Reliability plots for compressive strengths.

Table 12 presents the design residual compressive strength and residual strengths for the DPFRC under elevated temperature at different survival probabilities. The survival probabilities for the compressive strengths of the DPFRC are plotted and presented in Fig. 15a,b. The design life of the DPFRC should be selected in such a way that only a small probability that loss in compressive strength will occur under high temperature. From Fig. 15, as the survival probability increases both the residual compressive strength and relative strength decrease and vice versa. From Table 12 for instance, at 99% failure probability (1% survival probability), the design residual compressive strength and relative strengths are 3.05 MPa and 7.39% respectively. This implies that about 99% of the DPFRC mixes can survive subjecting to a temperature of up to 800 °C for 2 h duration without failure and still achieve a compressive strength of 3.05 MPa. Similarly, at 10% failure probability (90% survival probability), the design residual compressive strength and relative strength are 56.10 MPa and 149.51% respectively. Meaning, that about 10% of the DPFRC mixes can survive exposure to elevated temperature up to 800 °C for 2 h and still achieve a residual compressive strength up to 56.10 MPa without failure.

**Table 12:** Residual and relative strength at different survival probability.

Strengths at elevated temperature	Reliabilities/strengths				
	0.01	0.1	0.5	0.9	0.99
Residual compressive strength (MPa)	81.34	56.10	29.47	10.74	3.05
Relative strength (%)	219.42	149.51	76.94	27.13	7.39

## Conclusions

From the findings of the experimental results and analysis in this study. The following conclusions arises.

- The addition of DPF led to reduction in the residual compressive strength and relative strengths of the DPFRC, while the replacement of up to 10% cement with silica fume resulted to improvement in the residual compressive strength and relative strength of the DPFRC when subjected to a temperature up to 400 °C.
- The best validation performance for the model M-I is 0.000305017 at epoch 16 while for the model M-II is 0.0044732 at epoch 47.
- For neural networking model M-I, most of the predictive values carry errors in-between − 0.02 to + 0.02, which seems acceptable.
- The maximum predictive values by neural networking model M-II, carry error within bandwidth of − 0.05 to + 0.05
- For predictive neural model M-I, the regression is noticed R = 0.995 while for neural model M-II, the regression value is R = 0.989. Both depict the strong correlation between predicted and actual values.
- The multivariable regression models established for forecasting the performance of DPFRC exposed to high temperature have a very high statistical significance with a very good degree of correlation.
- The compressive strengths of the DPFRC concrete containing silica fume exposed to elevated temperature follows the two-parameter Weibull distribution functions.
- From the reliability analysis about 10% of the DFPRC mixes can survive exposure to elevated temperature up to 800 °C for 2 h and still achieve a compressive strength and relative strength up to 56.10 MPa and 149.51% respectively without failure.

For future works, the ANN techniques can be employed for the prediction of the mechanical properties and durability performance of PW concrete modified with GNP under normal environmental conditions. Additionally, the ANN techniques can be utilized for the prediction of the durability properties of the PW concrete modified with GNP subjected to other environmental conditions such as acid attack, salt or chloride attack, freeze and thaw.

## Data Availability

The datasets used and/or analyzed during the current study are available from the corresponding author on reasonable request. All data generated during this study are included in this published article.
